# Chicago Medical Society

**Published:** 1886-06

**Authors:** 


### CHICAGO MEDICAL SOCIETY.

Officially Reported for THE ATLANTA MEDICAL AND SURGICAL JOURNAL.

---

STATED MEETING, May 3, 1886.

The President, E. J. Doerring, M. D., in the chair.

Dr. M. P. Kossakowski exhibited a case of

### DOUBLE HARE-LIP AND CLEFT PALATE IN AN INFANT.

He promised a subsequent report of the case.

Dr. H. Gradle read a paper on

### PURE DRINKING WATER.

He said the only way of deciding the question whether the Chicago drinking water is ever deleterious to health is to search for the number and kinds of micro-organisms it contains. The sewerage is poured into Lake Michigan, the source of water supply, whenever the current in the river drifts towards the lake. It is well known that the discharges of typhoid fever—of which there are over 5,000 cases annually in the city—contain the typhoid bacilli. Any spores existing in them, and thus carried into the lake, are likely to remain alive in the water long enough for some few of them to enter the water mains. A similar danger is not unlikely in the case of other intestinal diseases, although the parasites causing them have not yet been identified. Such considerations lead one to suspect the water as the possible cause of infection, however rare.

The speaker has made some culture analyses according to the methods of Koch, and found from 1,000 to 1,500 living germs per cubic centimetre of water. Four to six varieties of bacilli are commonly met with in the water, mostly in the form of spores.

For purification the speaker recommended twenty minutes' boiling, or thorough filtration. Most filters in the market are inefficient. The Mallié ærifilter, however, was found to be practically bacteria-tight, when recently cleansed, though on continued use a few germs pass through it. It consists of a cylinder of porous clay screwed to the faucet, and surrounded by a glass cup, through which the water passes.

Dr. Long asked if it is demonstrated whether the bacteria in our drinking water comes from the sewerage or from the air.

Dr. L. Curtis said there is no doubt that water absorbs a great number of bacteria from the air, and that these may gain entrance into the circulation through the digestive tract as well as through the respiratory tract. He said there is no doubt of the possibility of malarial germs being introduced into the system by means of drinking water.

Dr. R. Tilley wished to know if it has been actually demonstrated that the bacillus tuberculosis finds its way through our sewers into the lake and drinking water. We are not justified in saying that our water contains this element of danger until it has been demonstrated.

Dr. J. Zeisler asked if Dr. Gradle had examined the Waukesha waters and found bacteria in them.

Dr. Gradle closed the discussion by saying he took pains not to state in his paper whether he thought the disease germs found in the water were introduced by means of the sewerage or air. It is not a vital point, so far as the use of the water is concerned, although if it is decided that these bacteria are introduced by means of the sewerage, and not the atmosphere, it would have a bearing on the question of where to dispose of our sewerage. However, there is no doubt but bacteria are introduced into large bodies of water by means of dust, vegetation and decayed leaves, and there is no reason to believe that bacteria found in our water are derived from the sewerage alone. A Russian chemist had examined the water found at St. Petersburg, and found that at a distance from the city the number of bacteria per cubic centimetre was considerably less than near the city. Dr. Gradle has examined water obtained from Lake Michigan about thirty-five miles from Chicago and had found about the same number of bacteria per cubic centimetre, but a less number of varieties than in city water. He believed the spores of typhoid and tuberculosis may find entrance into our lake water and become causes of disease in individuals who are not prepared to resist their attack. Dr. Gradle had never examined the Waukesha waters, but he had examined water from a spring on his own farm, which he believed to be as pure as Waukesha water, and had found only fifteen to twenty bacteria per drop and only three varieties. He believed that spring waters are the purest, as the bacteria they contain cannot be due to sewerage.

Dr. W. H. Lyford read a report of a case of

SCLERODERMA.

A girl, æt. 10 years, apparently healthy and of good family history. Five years ago she received a shock from a lightning stroke. Shortly afterwards her parents noticed, under the surface of the skin over the outer region of the left forearm, a delicately traced symmetrical figure, branching and ramifying in purplish colored lines with singular regularity. Dr. Lyford believed the lightning had paralyzed the cutaneous branches of the musculo-spiral nerve, and caused congestion of these filaments, with the marking as a result. In due course of time the skin has undergone the changes that take place in scleroderma. There is hyperæsthesia accompanied by pain, especially at night. The patch is also now slightly elevated above the surrounding healthy integument, and is only slightly movable, while at the elbow it is attached to the fascia and sheaths on the tendons, thus interfering somewhat with the movements of the forearm.

Dr. J. Zeisler said he had seen four cases of scleroderma, two of general scleroderma and two of partial scleroderma. The first two cases he saw in a clinic of Kaposi. Their appearance was striking. In scleroderma the features of the face are immobile, and therefore the patient cannot express the emotions. The skin and underlying tissues seem to grow together. The cases of partial scleroderma he had seen in this city. In one patient the patch of scleroderma was a ribbon-like band beyond one ear, pinkish in color, immovable upon the underlying tissues. The other case was a lady, æt. 50 years, who noticed her right mammary gland was becoming hard, large, and the nipple contracted. There was no pain in the breast, nor were the axillary glands involved. The skin was hard and adherent to underlying tissue.

A similar condition was observed in a patch of skin on the right arm. Kaposi states the majority of cases of scleroderma occur in females. The best treatment for scleroderma is massage and galvanic electricity.

Dr. H. Gradle presented some stereoscopic views of sections of hypertrophied tonsils taken from Troutman's book on "Hypertrophied Tonsils," after which the Society adjourned.

---

CANCER OF THE STOMACH.—E. Hahn, in a paper on cancer of the stomach and operative treatment, gives the following valuable data :

Out of 7,205 post-mortem examinations, 166 were cases of carcinoma ventriculi, or 2.3 per cent. The cases were analyzed by the author with regard to the anatomical position of the disease, the question of metastasis, the sex and age of the patient. The result showed that of 166 cases, 98 were males and 68 females. One-half of the whole number were over sixty years of age, and only 2 were under thirty. With reference to the posi-position of the cancerous product, in 60 cases it was at the pyloric, in 40 at the cardiac extremity, in 27 in the lesser curvature, in 21 involving the whole surface, in 8 on the greater curvature, in 7 on the anterior wall, and in 3 on the posterior wall. In 29 cases, or nearly one-half of the cases where the pylorus was affected, there was metastasis, partly to the epigastric lymphatic glands and partly to the liver. In only 8 of these cases was there a complete absence of metastasis. In the other cases there seemed to be a still greater disposition to metastasis. Out of the 166 cases, 27 were of the scirrhus, 75 ulcerating, and 7 of the colloid variety. The last variety showed a special disposition to give metastasis to the peritoneum.—*Centralblatt*, *f. Chirurg.*, *May 8*, *1886*.

